# Impact of tranexamic acid on hidden blood loss in intertrochanteric fractures: a meta-analysis of randomized controlled trials

**DOI:** 10.3389/fsurg.2025.1681209

**Published:** 2025-11-24

**Authors:** Yan Meng, Yong Zhang, Hanzhong Xue, Ning Duan, Zhong Li, Qian Wang, Yao Lu

**Affiliations:** 1 Department of Surgery, Honghui Hospital, Xi’an Jiaotong University, Xi’an, Shaanxi, China; 2 Department of Joint Surgery, Beidaihe Rest and Recuperation Center of PLA, Qinhuangdao, Hebei, China; 3 Department of Orthopaedic Surgery, Honghui Hospital, Xi’an Jiaotong University, Xi’an, Shaanxi, China

**Keywords:** intertrochanteric fractures, tranexamic acid, hidden blood loss, hip fractures, mortality

## Abstract

**Background:**

Intertrochanteric fractures (IFs) are a common type of fracture in the elderly and are often associated with substantial hidden blood loss (HBL) due to trauma and surgery. Tranexamic acid (TXA) has emerged as a potential intervention to reduce perioperative bleeding. This study aimed to evaluate the safety and efficacy of TXA administration in elderly patients with IFs undergoing intramedullary nailing, through a systematic review and meta-analysis of randomized controlled trials (RCTs).

**Methods:**

Web of Science, Cochrane Library, Embase, and PubMed were searched for relevant RCTs published from inception to January 2025. Data on HBL, total blood loss (TBL), transfusion rate, and thromboembolic events were extracted. Review Manager 5.3.5 was used to assess the safety and efficacy of TXA.

**Results:**

Eight RCTs involving 735 patients (363 in the TXA group and 372 in the control group) were included in the meta-analysis. The TXA group demonstrated significantly lower HBL [standard mean difference (SMD) = −0.59; 95% confidence interval (CI), −0.74 to −0.45] and TBL (SMD = −0.74; 95% CI, −0.91 to −0.58), as well as a reduced transfusion rate [relative risk (RR) = 0.50; 95% CI, 0.35–0.72] compared with the control group. Additionally, no significant difference in thromboembolic events was found between the two groups.

**Conclusions:**

Current evidence indicates that TXA significantly reduces HBL and transfusion requirements without increasing the risk of thromboembolic events in elderly patients with IFs.

## 1 Introduction

Intertrochanteric fractures (IFs) are a common type of fracture in the elderly, often resulting from falls or trauma. These injuries significantly impair quality of life and increase the risk of long-term complications and healthcare costs, particularly in aging societies (1). Current treatment modalities for IFs include surgical fixation techniques such as intramedullary nailing. However, despite advancements in surgical techniques, patients frequently experience perioperative complications, including hidden blood loss, which can increase the need for blood transfusion and adversely affect recovery (2). Our previous study reported 1-year, 2-year, and 3-year mortality rates after IF surgery of 9.6%, 16.7%, and 24.4%, respectively (3). Perioperative anemia is an important risk factor for mortality following IF surgery (3, 4). Allogeneic blood transfusion is a common method used to treat severe anemia. However, it carries a higher risk of transfusion reactions and bacterial infection (5).

Tranexamic acid (TXA) is an antifibrinolytic agent that has received increasing attention in recent years for its potential to reduce perioperative blood loss in various surgical settings (6–8). Numerous studies have suggested that TXA administration may significantly reduce intraoperative and postoperative blood loss and lower transfusion requirements, without increasing the risk of thromboembolic complications during hip and knee replacement surgeries (8, 9). Most studies have focused on hip fractures, which include both IFs and femoral neck fractures. However, important distinctions exist between these two types of fractures, including differences in patient demographics, surgical interventions, and prognosis. Many questions remain regarding the efficacy and safety of TXA in elderly patients with IFs undergoing intramedullary nailing.

Therefore, the aim of this meta-analysis was to assess the effectiveness and safety of TXA in patients undergoing IF surgery with intramedullary nailing.

## 2 Materials and methods

This systematic review and meta-analysis was conducted in accordance with the Preferred Reporting Items for Systematic Reviews and Meta-Analyses (PRISMA) guidelines (10). As no individual participant data were collected, additional ethical approval was not required.

### 2.1 Search strategy

The electronic databases Web of Science, Cochrane Library, Embase, and PubMed were searched for relevant randomized controlled trials (RCTs) published from database inception to January 2025. Two independent researchers conducted the literature search using the following keywords: “Tranexamic Acid”, “TXA”, “Intertrochanteric Fractures”, “Subtrochanteric Fractures”, “proximal femoral nail anti-rotation”, “PFNA”, “intramedullary nailing”, and “IMN”. The search strategy is shown in Supplementary Material 1. In addition, the reference lists of relevant reviews were screened to identify additional studies eligible for inclusion in the meta-analysis.

### 2.2 Eligibility criteria

The inclusion criteria for this study were as follows: (1) adult patients diagnosed with intertrochanteric fractures; (2) intervention group receiving intravenous (IV) TXA treatment; (3) control group receiving placebo, saline, or no intervention; (4) study design as a randomized controlled trial (RCT); (5) undergoing IF surgery with intramedullary nailing; and (6) reporting of hidden blood loss as an outcome indicator. The exclusion criteria were: (1) reviews, conference abstracts, commentaries, or other non-original research articles; (2) animal studies; (3) retrospective studies, cohort studies, case reports, and case series; and (4) duplicate publications or multiple reports based on the same dataset, in which case only the study with the most comprehensive information was included.

#### 2.2.1 Data extraction and quality assessment

Two investigators independently extracted data according to the predefined inclusion and exclusion criteria. The extracted information included the first author's name and publication year, study design, basic characteristics of the study population (sample size, age, and gender composition), fixation method, intervention details, follow-up duration, and outcomes [total blood loss (TBL), hidden blood loss (HBL), transfusion rate, and thromboembolic events]. After completing the data extraction, the investigators exchanged and reviewed their extraction tables. Any discrepancies were resolved through discussion. The Cochrane Collaboration's risk of bias tool was used to assess the quality of the included RCTs (11).

#### 2.2.2 Statistical analysis

Statistical analyses were performed using RevMan version 5.3.5 and Stata version 14.0. For continuous data, the standard mean difference (SMD) with 95% confidence intervals (CIs) was calculated. For dichotomous data, relative risk (RR) with 95% CIs was used. Heterogeneity among studies was assessed using the chi-square test and the I^2^ statistic. A fixed-effects model was applied when *I*^2^ ≤ 50%, while a random-effects model was used when *I*^2^ > 50%. Egger's test was conducted to evaluate the presence of significant publication bias.

## 3 Results

### 3.1 Included studies

**Figure 1:**
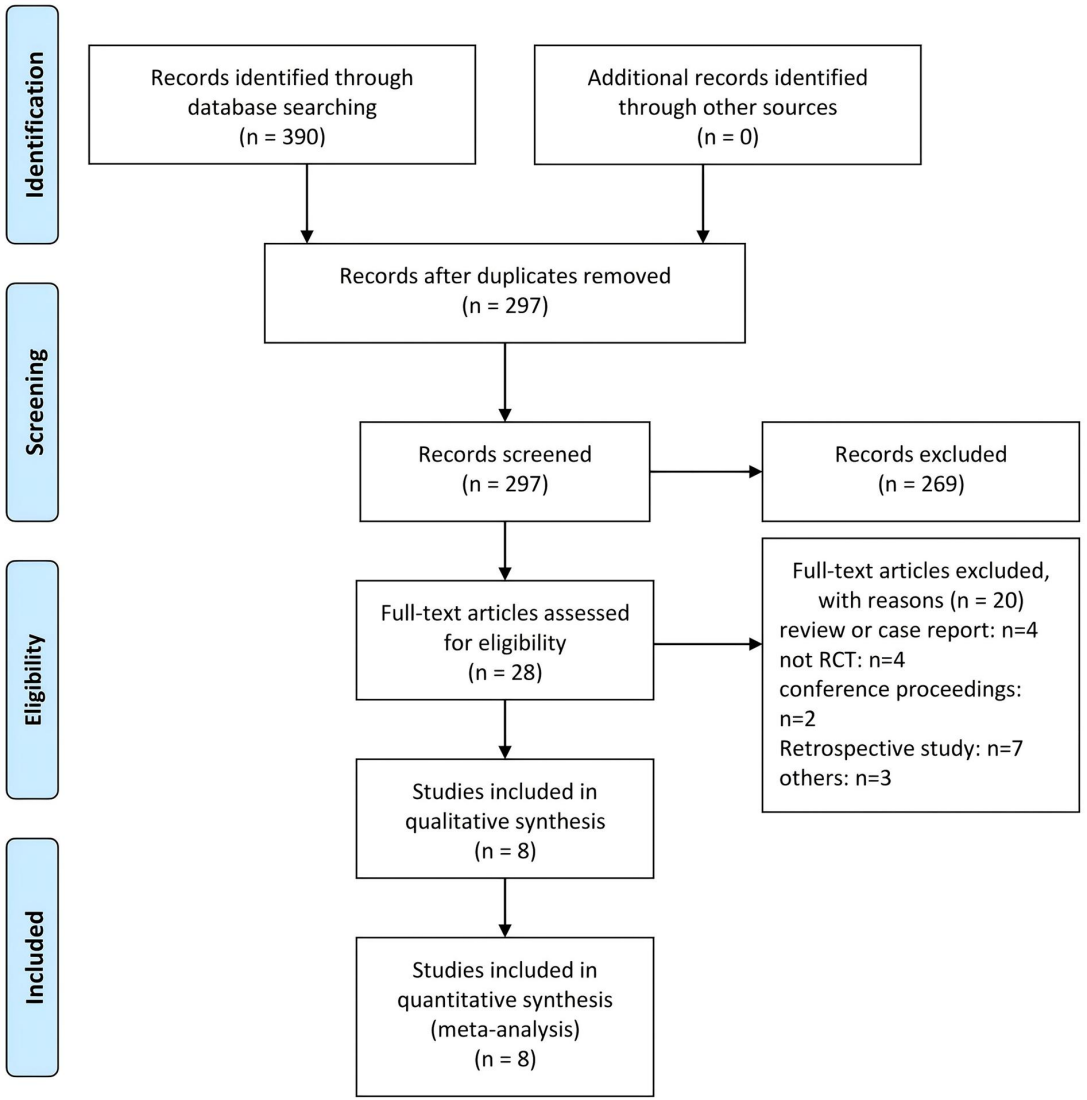
PRISMA flowchart.

The results of the literature search and screening process are presented in Figure 1. A total of 390 articles were retrieved from electronic databases (PubMed: 45; Embase: 58; Web of Science: 287). After removing 93 duplicates, 297 records remained. Screening of titles and abstracts led to the exclusion of 269 articles that did not meet the inclusion criteria. Full-text reviews were then conducted on the remaining 28 articles, of which 20 were excluded. Ultimately, 8 articles were included in the meta-analysis (12–19). Out of the 8 RCTs included in this study, 4 have clinical trial registration numbers (16–19).

### 3.2 Study characteristics and quality evaluation

**Figure 2:**
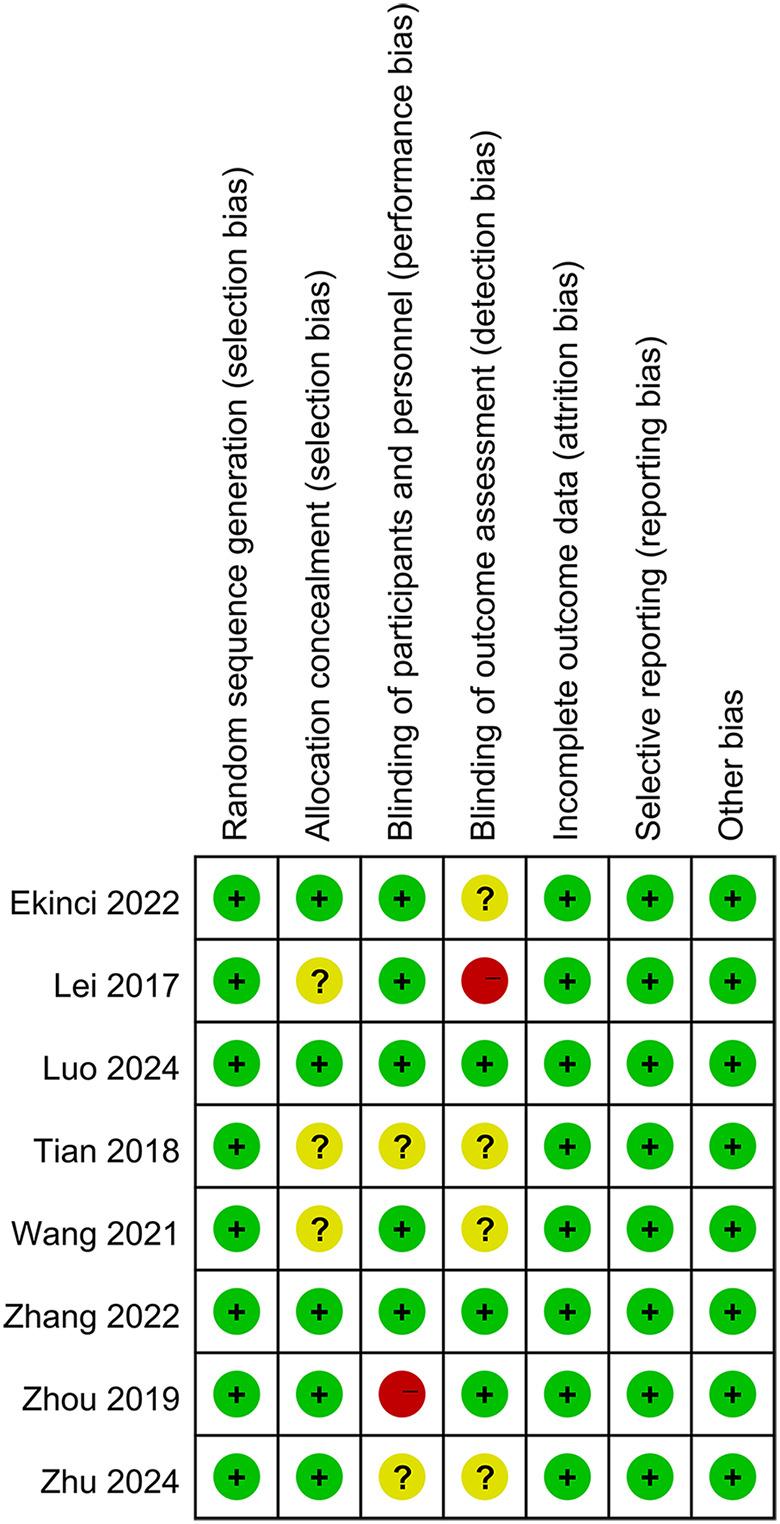
The risk of bias for each study.

Eight RCTs comprising a total of 735 patients were included in the analysis, with sample sizes ranging from 55 to 122 patients. Among these, 363 patients were in the TXA group and 372 in the control group. The majority of participants (62.31%) were female, with an age range of 72–83 years. All patients were diagnosed with intertrochanteric fractures and underwent closed reduction and internal fixation using intramedullary nails. All included studies administered TXA via the IV route (Table 1). The quality of the included studies was rated as moderate to high. A summary of the risk of bias for each study is presented in Figure 2.

**Table 1:** The characteristics of the included patients.

Study	Sample size (TXA/CON)	Gender (M/W)		Ages (years)		Fixation method	Intervention		Follow up (months)
		TXA	CON	TXA	CON		TXA	CON	
Lei 2017	37/40	5/32	7/33	77.80 ± 9.75	79.18 ± 6.50	PFNA	200 ml (1 g) of TXA i.v. before surgery	Normal-saline	1 month
Tian 2018	50/50	19/31	14/36	77.74 ± 6.53	79.25 ± 6.55	PFNA	TXA (10 mg/kg-1) i.v. 10 min preoperatively and 5 h postoperatively	Blank control	
Zhou 2019	50/50	15/35	22/28	75.10 ± 8.27	77.82 ± 6.42	PFNA	TXA (1 g/100 ml) i.v. before surgery	Blank control	1 month
Wang 2021	33/32	10/22	10/23	72.25 ± 7.65	75.15 ± 9.36	PFNA	TXA (1 g) i.v. before surgery	Physiological saline	
Ekinci 2022	51/51	26/25	19/32	76.0 ± 18.3	79.8 ± 10.5	PFNA	TXA (15 mg/kg) i.v. before the incision and after anesthesia	Saline	3 months
Zhang 2022	61/61	28/33	34/27	79.11 ± 11.91	76.07 ± 16.60	PFNA	TXA (1 g) i.v. 10 min before incision and 3 h later	Normal saline	3 months
Luo 2024	56/56	25/31	17/39	80.59 ± 8.11	83.41 ± 7.91	PFNA	TXA (1.5 g) i.v. before surgery	Normal saline	3 months
Zhu 2024	25/32	15/10	11/21	77.60 ± 9.96	80.05 ± 9.31	Intramedullary nailing	TXA (1 g) i.v. before surgery	Blank control	12 months

TXA, tranexamic acid; CON, control; M/F, male/female; PFNA, proximal femoral nail antirotation.

### 3.3 Meta-analysis results

#### 3.3.1 HBL

**Figure 3:**
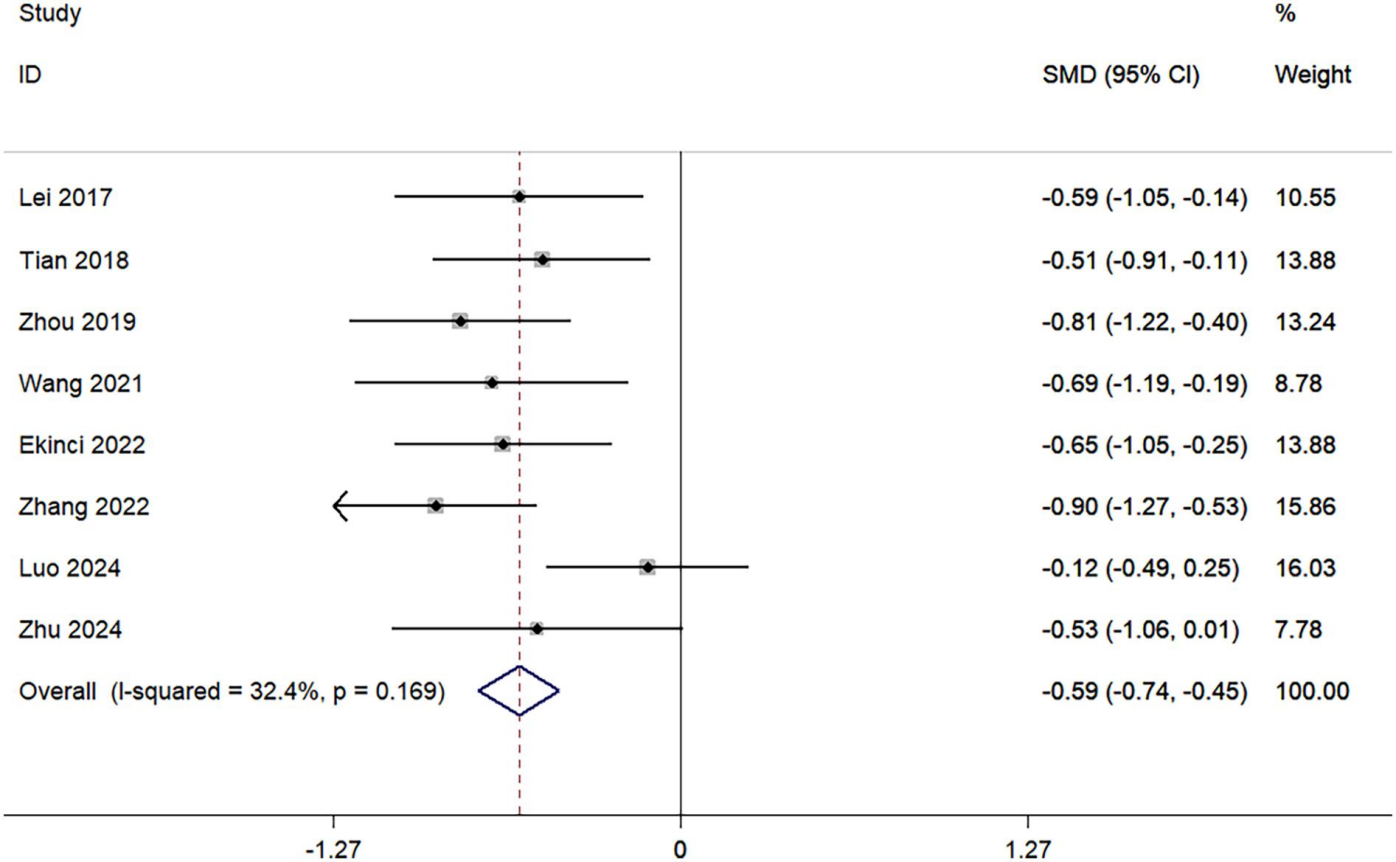
Forest plot for hidden blood loss.

A total of eight studies reported outcomes on HBL. There was no significant heterogeneity among these studies (I^2^ = 32.4%, *p* = 0.169); therefore, a fixed-effects model was used for the analysis. The pooled results indicated that the amount of HBL in the TXA group was significantly lower than in the control group (SMD = −0.59; 95% CI, −0.74 to −0.45; Figure 3).

#### 3.3.2 TBL

**Figure 4:**
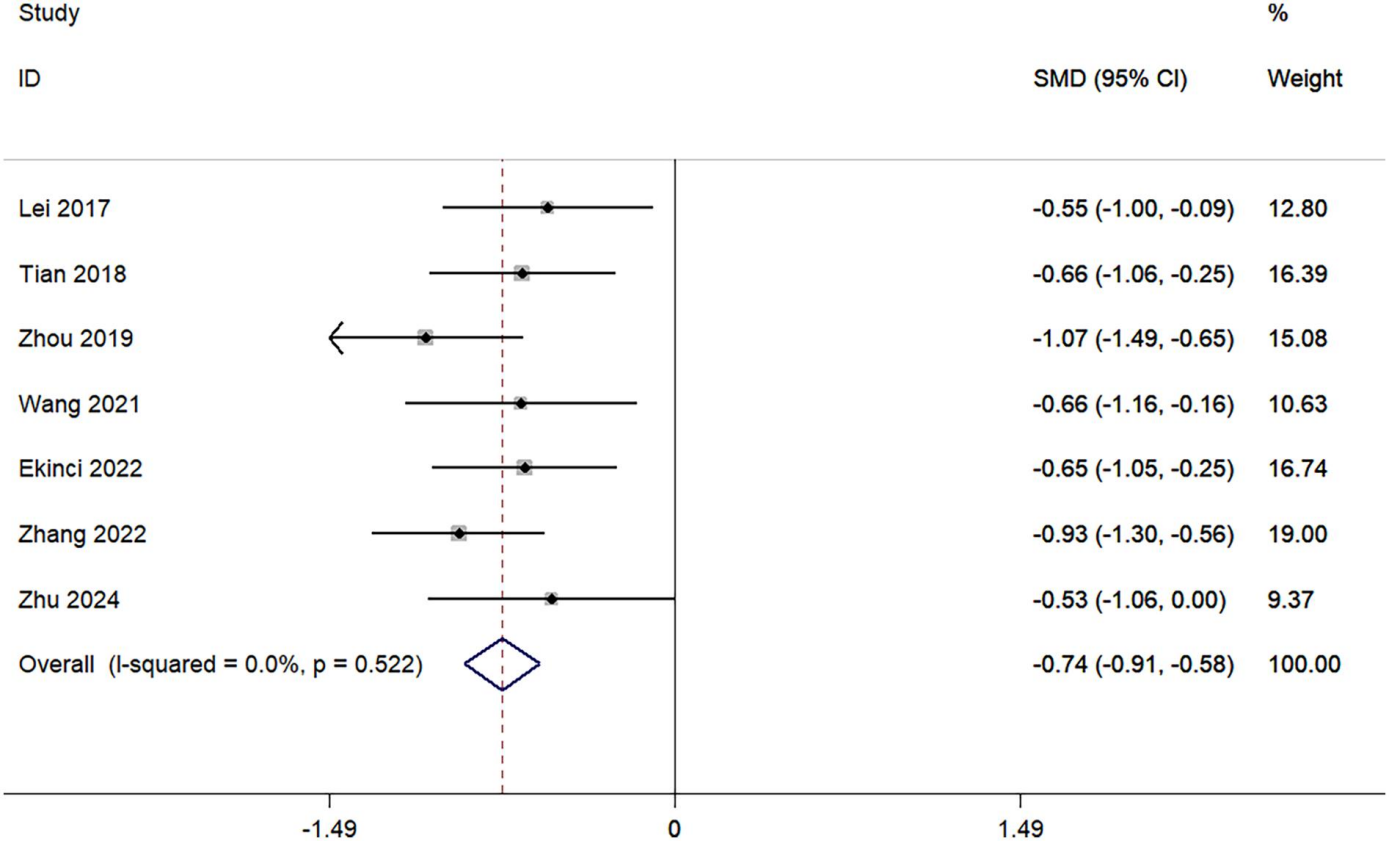
Forest plot for total blood loss.

Seven studies investigated TBL. No heterogeneity was observed among the studies (I^2^ = 0.0%, *p* = 0.522), and a fixed-effects model was applied. The pooled data showed that TBL in the TXA group was significantly lower than in the control group (SMD = −0.74; 95% CI, −0.91 to −0.58; Figure 4).

#### 3.3.3 Transfusion rate

**Figure 5:**
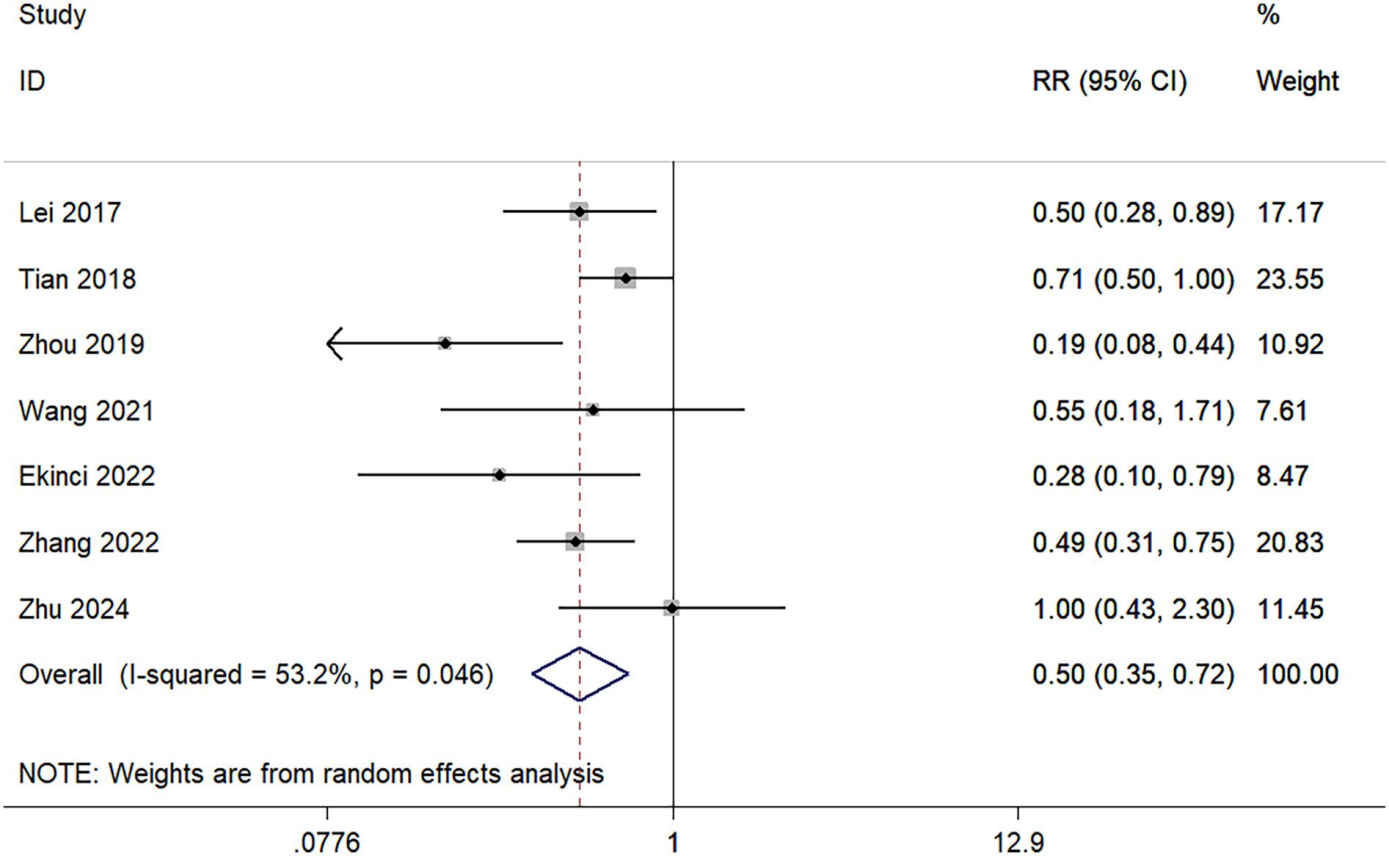
Forest plot for transfusion rate.

Transfusion rate outcomes were reported in seven studies. The transfusion rate was 27.4% (84/307) in the TXA group and 51.6% (163/316) in the control group. Significant heterogeneity was identified in the pooled analysis (I^2^ = 53.2%, *p* = 0.046); thus, a random-effects model was used. The pooled results demonstrated that the transfusion rate in the TXA group was significantly lower than in the control group (RR = 0.50; 95% CI, 0.35–0.72; Figure 5).

#### 3.3.4 Thromboembolic events

**Figure 6:**
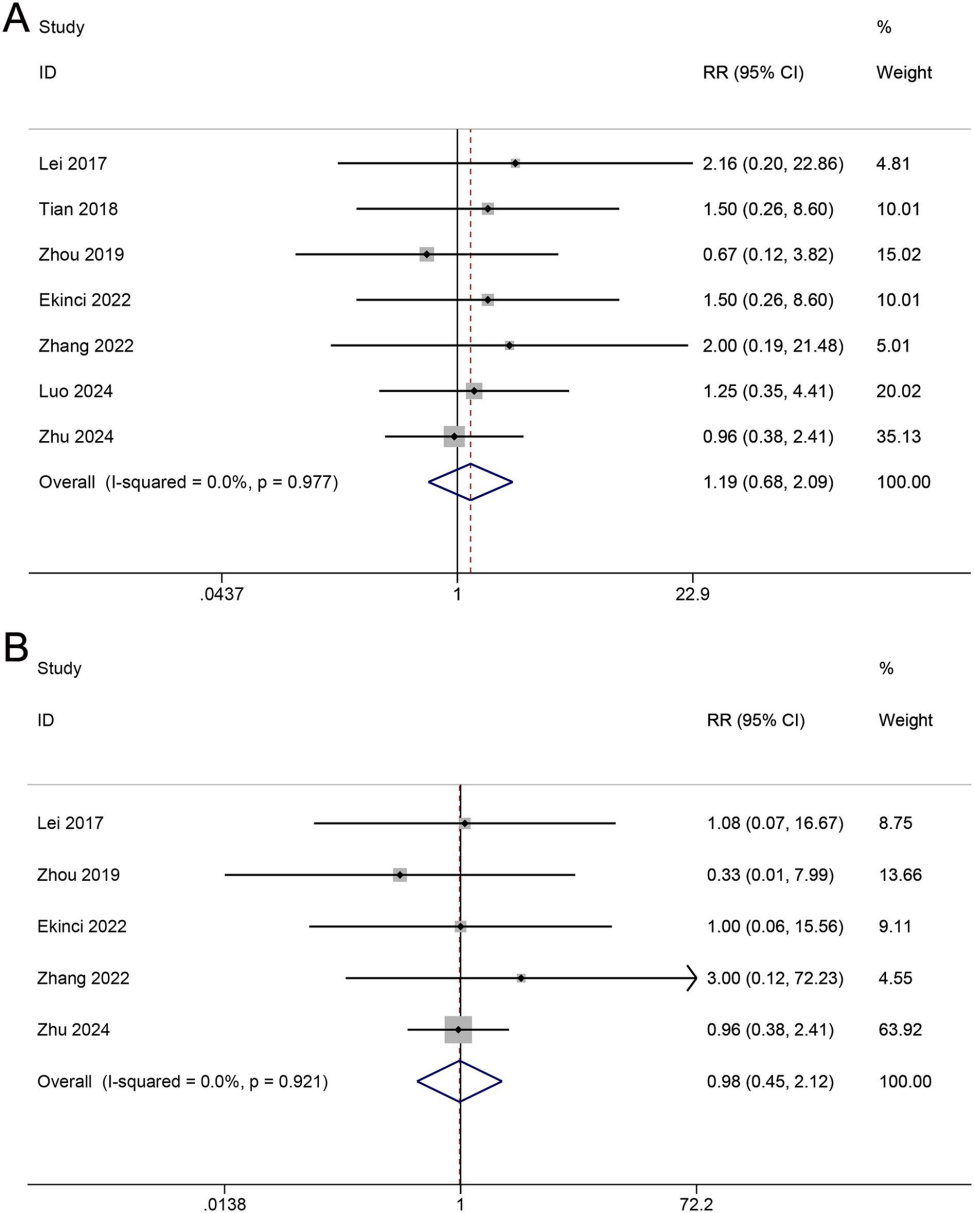
Forest plot for thromboembolic events. **(A)** DVT. **(B)** PE.

Thromboembolic events include lower-limb deep vein thrombosis (DVT) and pulmonary embolism (PE). Seven studies reported data on DVT events. The incidence of lower-limb DVT in the TXA group was 6.9% (23/330), while it was 6.1% (21/340) in the control group. No heterogeneity was observed among the studies (I^2^ = 0.0%, *p* = 0.977), and a fixed-effects model was used for the analysis. The meta-analysis indicated that there was no significant difference in DVT events between the TXA and control groups (RR = 1.19; 95% CI, 0.68–2.09; Figure 6A). Five studies reported data on PE events. The incidence of PE in the TXA group was 6.9% (9/224), while it was 6.1% (11/234) in the control group. No heterogeneity was observed among the studies (I^2^ = 0.0%, *p* = 0.921), and a fixed-effects model was used for the analysis. The meta-analysis indicated that there was no significant difference in PE events between the TXA and control groups (RR = 0.98; 95% CI 0.45–2.12; Figure 6B).

#### 3.3.5 Sensitivity analysis and publication bias

**Figure 7:**
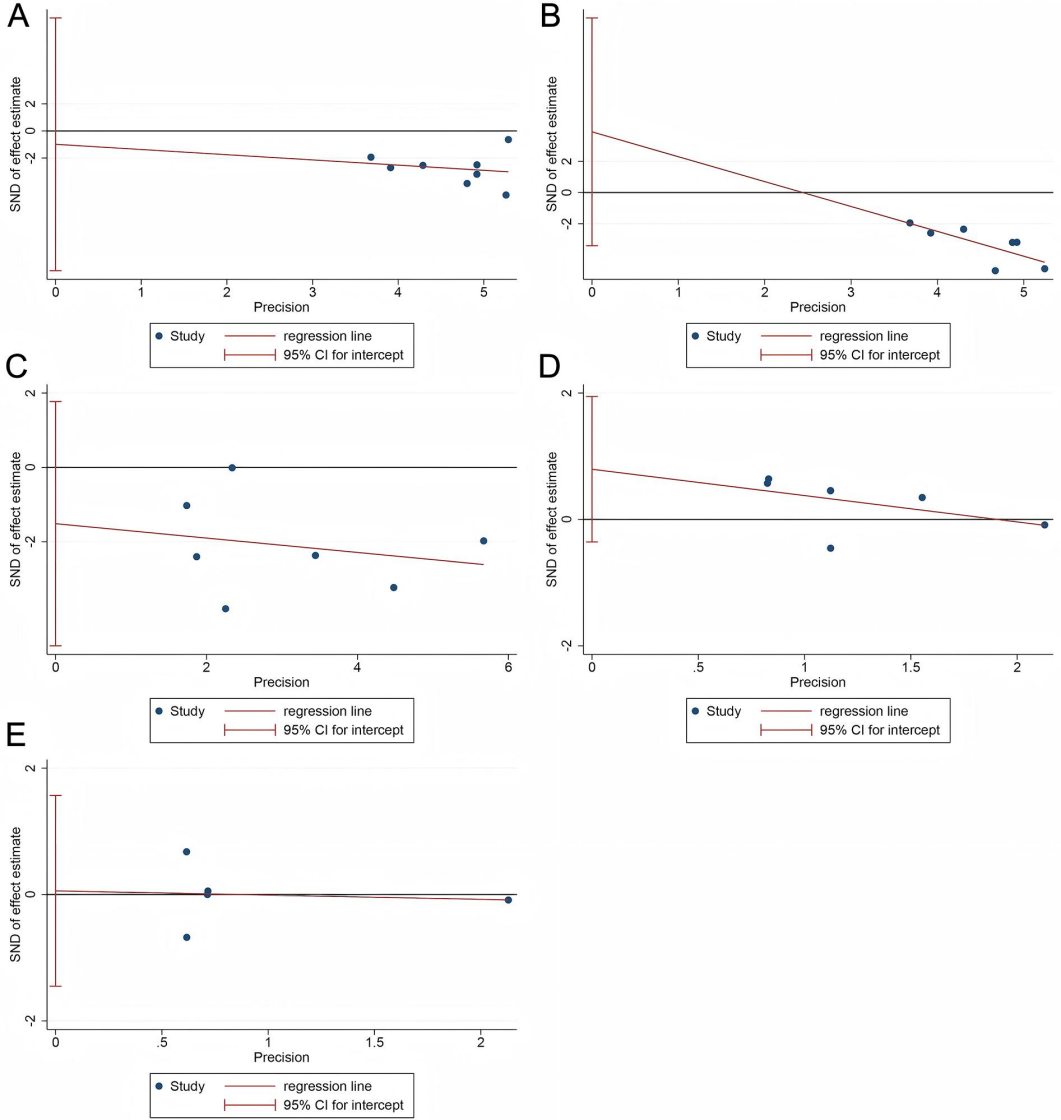
Publication bias. **(A)** HBL; **(B)** TBL; **(C)** Transfusion rate; **(D)** DVT; **(E)** PE.

As shown in Figure 7, the results of the Egger test for all four outcomes—HBL (*p* = 0.805), TBL (*p* = 0.229), transfusion rate (*p* = 0.08), DVT (*p* = 0.289) and PE (*p* = 0.911) — were not statistically significant (all *p* > 0.05), indicating no evidence of significant publication bias.

**Figure 8:**
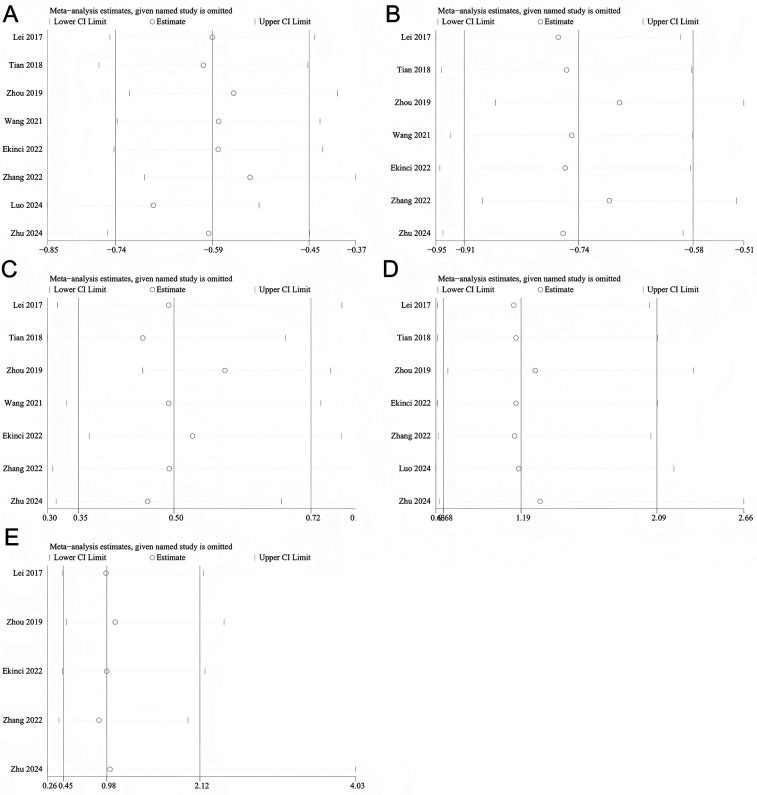
Sensitivity analysis. **(A)** HBL; **(B)** TBL; **(C)** Transfusion rate; **(D)** DVT; **(E)** PE.

The results of the sensitivity analysis indicated that excluding any single study did not significantly affect the pooled SMD (HBL, TBL, transfusion rate, DVT and PE) (Figure 8), suggesting that the findings of this meta-analysis are relatively robust.

## 4 Discussion

IF is a common injury among the elderly, often resulting from falls or traumatic incidents. These fractures significantly impair the quality of life of affected individuals and increase the risk of long-term complications, including prolonged immobility and elevated healthcare costs (1). As the global population ages, the incidence of IF is expected to rise, making effective treatment and prevention strategies increasingly important (1). Current management options primarily include surgical stabilization; however, challenges such as postoperative bleeding, hematoma formation, and delayed recovery persist (20–22). Therefore, exploring new therapeutic approaches—such as the use of antifibrinolytic agents like TXA—to reduce postoperative blood loss is crucial for improving outcomes in this population (22–24).

The present study aimed to provide high-quality evidence on the efficacy and safety of intravenous TXA administration in patients with IF undergoing intramedullary nailing, through a systematic review and meta-analysis of randomized controlled trials. The primary findings of this study are as follows: (1) intravenous TXA was associated with significantly lower HBL, TBL, and transfusion rates compared to the control group; and (2) there was no significant difference in the incidence of thromboembolic events between the TXA and control groups. These results provide clinically relevant evidence supporting the use of TXA in IF management and may inform future clinical practice and research in this area.

HBL is a significant yet often underestimated phenomenon observed in patients undergoing surgical procedures, particularly in the context of IF (2). HBL refers to the blood loss that is not readily apparent or directly measured during surgery, in contrast to overt blood loss, which is visibly identifiable and quantifiable. HBL may result from various physiological and surgical factors and can lead to a substantial decline in hemoglobin levels without obvious external bleeding. Although the intramedullary nailing technique is a widely used treatment for IF and effectively reduces intraoperative blood loss due to its minimally invasive nature and stable fixation, it is still associated with considerable total perioperative blood loss—averaging 911.3 ml, with approximately 84.5% classified as HBL (22). Furthermore, HBL after intramedullary nailing for intertrochanteric fractures has been shown to exceed intraoperative blood loss significantly (15, 25). Perioperative anemia is an important risk factor for mortality following IF surgery (3, 4). Postoperative anemia exacerbates underlying health conditions, delays functional recovery, and increases postoperative mortality rates (26, 27). Patients with postoperative anemia often present with multiple comorbidities, such as congestive heart failure and chronic anemia, which significantly limit their ability to tolerate any compensatory decline in oxygen-carrying capacity. This context is crucial for understanding the impact of postoperative anemia on mortality in these patients (3, 4, 26, 27).

TXA is an antifibrinolytic agent that acts by binding to the lysine-binding sites of plasminogen, thereby inhibiting the breakdown of fibrin clots. TXA is frequently used in orthopedic procedures, particularly in joint arthroplasty (6, 28). Despite its well-established cost-effectiveness in minimizing blood loss during elective surgical procedures, concerns remain regarding its efficacy and, more importantly, its safety in the context of fracture repair (29). Previous research has largely focused on the use of TXA in various surgical fields; however, its specific application in IF has not been adequately addressed (30–33). Our prior study also demonstrated that a sequential intravenous TXA regimen in elderly patients with IF undergoing surgery helped maintain hemoglobin levels and reduce transfusion rates (34). In the present meta-analysis, the amount of HBL in the TXA group was significantly lower than in the control group (SMD = −0.59; 95% CI, −0.74 to −0.45). The pooled results also showed that TBL in the TXA group was significantly lower than in the control group (SMD = −0.74; 95% CI, −0.91 to −0.58). The transfusion rate in the TXA group was 27.4% (84/307), compared with 51.6% (163/316) in the control group. The pooled results demonstrated that the transfusion rate in the TXA group was significantly lower than in the control group (RR = 0.50; 95% CI, 0.35–0.72). This study represents a meaningful advancement in understanding the role of TXA in reducing HBL among elderly patients with IF. Our findings show that TXA not only significantly reduces hidden and total blood loss but also decreases the need for blood transfusion. These findings are consistent with a previous meta-analysis by Jiakai Zhang et al. (24), which similarly found no increased risk of adverse events associated with transfusion.

Although evidence supports the efficacy of TXA in reducing blood loss, concerns have been raised regarding a potential increase in thrombotic events. One report noted a vascular event rate of 16% in the TXA group vs. 6% in the placebo group within six weeks postoperatively (35). However, the difference was not statistically significant (35). A randomized controlled trial by Gang Luo et al. (16) similarly reported no significant difference in the incidence of thromboembolic events between groups receiving repeated intravenous doses of TXA in elderly patients with IF. Additionally, a recent meta-analysis (36) involving 1,397 patients with IF—699 of whom received intravenous TXA and 698 received normal saline—concluded that TXA administration was safe and did not increase the risk of thromboembolic events. In our study, the incidence of DVT in the lower limbs was 7.4% (27/353) in the TXA group and 6.5% (24/372) in the control group. The pooled analysis showed no significant difference in thromboembolic events (DVT, RR = 1.19; 95% CI, 0.68–2.09 and PE, RR = 0.98; 95% CI 0.45–2.12) between the TXA and control groups. These findings are consistent with previous study results.

Furthermore, our results corroborate the findings of Veronique et al. (37), who reported that TXA effectively reduced blood loss in patients undergoing hip arthroplasty, thereby reinforcing its potential benefits in geriatric populations. The implications of our findings extend to both clinical practice and policy development in the management of intertrochanteric fractures. The significant reductions in hidden blood loss and transfusion rates suggest that TXA may serve as a valuable adjunct therapy in this patient population, potentially improving postoperative outcomes and lowering healthcare costs associated with transfusion. Incorporating TXA into standard surgical protocols for elderly patients with intertrochanteric fractures may enhance recovery trajectories and mitigate complications related to perioperative blood loss, which are particularly concerning in this vulnerable population (38, 39). These findings support the broader adoption of TXA in clinical guidelines and promote evidence-based practices aimed at improving patient outcomes and optimizing healthcare resource utilization.

### 4.1 Limitations

This study is not without limitations. Although the analysis incorporated predominantly high-quality, level I evidence, the relatively small sample size—eight RCTs comprising 735 patients—may limit the generalizability of our findings. Additionally, some studies included follow-up durations of only one month, providing insufficient data to evaluate long-term outcomes. The short follow-up period restricts our ability to assess delayed complications associated with TXA use. Future research should aim to conduct larger, multicenter trials with extended follow-up to further validate the safety and efficacy of TXA in this clinical context. Furthermore, in the current meta-analysis, patients in the intervention group received intravenous TXA, but the optimal dosage and timing of administration to minimize blood loss remain unclear. Regarding the possibility of a sub-group analysis, we initially considered this; however, the sample size and the variability in patient demographics and surgical procedures limited our ability to conduct a meaningful sub-group analysis. The type of IF and the experience and expertise of the operating surgeon can influence per operative blood loss in IF. Despite these constraints, our analysis incorporated the most recent high-quality RCTs, and the findings were supported by robust statistical evidence.

In conclusion, this meta-analysis demonstrates that TXA significantly reduces hidden blood loss, total blood loss, and transfusion requirements in patients with intertrochanteric femoral fractures, without increasing the risk of thromboembolic events. These findings support the clinical use of TXA as an effective strategy for minimizing perioperative blood loss and transfusion dependency. However, further investigation is warranted to assess TXA's efficacy across different patient subgroups and treatment strategies, thereby strengthening its role in improving surgical outcomes in this vulnerable geriatric population.

## Data Availability

The original contributions presented in the study are included in the article/Supplementary Material, further inquiries can be directed to the corresponding authors.
