# Supplementary material for: Impact of tranexamic acid on hidden blood loss in intertrochanteric fractures: a meta-analysis of randomized controlled trials
Source: Front Surg. 2025 Nov 24;12:1681209. doi: 10.3389/fsurg.2025.1681209 (PMC12682745; doi:10.3389/fsurg.2025.1681209)
Supplement: Supplementary file 1 [file Supplementaryfile1.docx]

**Pubmed：**

(((((((((((((tranexamic acid[Title/Abstract]) OR (AMCA[Title/Abstract])) OR (AMCHA[Title/Abstract])) OR (t-AMCHA[Title/Abstract])) OR (trans-4-(Aminomethyl)cyclohexanecarboxylic Acid[Title/Abstract])) OR (Cyklokapron[Title/Abstract])) OR (Ugurol[Title/Abstract])) OR (Transamin[Title/Abstract])) OR (KABI 2161[Title/Abstract])) OR (Amchafibrin[Title/Abstract])) OR (Anvitoff[Title/Abstract])) OR (Spotof[Title/Abstract])) OR (Exacyl[Title/Abstract])) AND ((((((((((((((((((((intertrochanteric fracture[Title/Abstract]) OR (Fractures, Hip[Title/Abstract])) OR (Intertrochanteric Fractures[Title/Abstract])) OR (Fractures, Intertrochanteric[Title/Abstract])) OR (Subtrochanteric Fractures[Title/Abstract])) OR (Fractures, Subtrochanteric[Title/Abstract])) OR (Trochanteric Fractures[Title/Abstract])) OR (Fractures, Trochanteric[Title/Abstract])) OR (Trochlear Fractures, Femur[Title/Abstract])) OR (Femur Trochlear Fracture[Title/Abstract])) OR (Femur Trochlear Fractures[Title/Abstract])) OR (Fracture, Femur Trochlear[Title/Abstract])) OR (Fractures, Femur Trochlear[Title/Abstract])) OR (Trochlear Fracture, Femur[Title/Abstract])) OR (Femoral Trochlear Fractures[Title/Abstract])) OR (Femoral Trochlear Fracture[Title/Abstract])) OR (Fracture, Femoral Trochlear[Title/Abstract])) OR (Fractures, Femoral Trochlear[Title/Abstract])) OR (Trochlear Fracture, Femoral[Title/Abstract])) OR (Trochlear Fractures, Femoral[Title/Abstract]))

**Web of science：**

(((tranexamic acid) OR (AMCA) OR (AMCHA) OR (t-AMCHA) OR (trans-4-(Aminomethyl)cyclohexanecarboxylic Acid) OR (Cyklokapron) OR (Ugurol) OR (Transamin) OR (KABI 2161) OR (Amchafibrin) OR (Anvitoff) OR (Spotof) OR (Exacyl)) AND ((intertrochanteric fracture) OR (Fractures, Hip) OR (Intertrochanteric Fractures) OR (Fractures, Intertrochanteric) OR (Subtrochanteric Fractures) OR (Fractures, Subtrochanteric) OR (Trochanteric Fractures) OR (Fractures, Trochanteric) OR (Trochlear Fractures, Femur) OR (Femur Trochlear Fracture) OR (Femur Trochlear Fractures) OR (Fracture, Femur Trochlear) OR (Fractures, Femur Trochlear) OR (Trochlear Fracture, Femur) OR (Femoral Trochlear Fractures) OR (Femoral Trochlear Fracture) OR (Fracture, Femoral Trochlear) OR (Fractures, Femoral Trochlear) OR (Trochlear Fracture, Femoral) OR (Trochlear Fractures, Femoral)))

**Embase:**

#33. #14 AND #32

#32. #15 OR #16 OR #17 OR #18 OR #19 OR #20 OR #21 OR #22 OR #23 OR #24 OR #25 OR #26 OR #27 OR #28 OR #29 OR #30 OR #31

#31. 'trochlear fractures, femoral':ab,ti

#30. 'trochlear fracture, femoral':ab,ti

#29. 'fractures, femoral trochlear':ab,ti

#28. 'femoral trochlear fractures':ab,ti

#27. 'trochlear fracture, femur':ab,ti

#26. 'fractures, femur trochlear':ab,ti

#25. 'femur trochlear fractures':ab,ti

#24. 'femur trochlear fracture':ab,ti

#23. 'trochlear fractures, femur':ab,ti

#22. 'fractures, trochanteric':ab,ti

#21. 'trochanteric fractures':ab,ti

#20. 'fractures, subtrochanteric':ab,ti

#19. 'subtrochanteric fractures':ab,ti

#18. 'fractures, intertrochanteric':ab,ti

#17. 'intertrochanteric fractures':ab,ti

#16. 'fractures, hip':ab,ti

#15. 'intertrochanteric fracture':ab,ti

#14. #1 OR #2 OR #3 OR #4 OR #5 OR #6 OR #7 OR #8 OR #9 OR #10 OR #11 OR #12 OR #13

#13. 'exacyl':ab,ti

#12. 'spotof':ab,ti

#11. 'anvitoff':ab,ti

#10. 'amchafibrin':ab,ti

#9. 'kabi 2161':ab,ti

#8. 'transamin':ab,ti

#7. 'ugurol':ab,ti

#6. 'cyklokapron':ab,ti

#5. 'trans-4-(aminomethyl)cyclohexanecarboxylic acid':ab,ti

#4. 't-amcha':ab,ti

#3. 'amcha':ab,ti

#2. 'amca':ab,ti

#1. 'tranexamic acid':ab,ti
